# Tethelin

**Published:** 1919-02-15

**Authors:** 


### TETHELIN.

One of the most interesting and potent of the ductless glands is the pituitary body, a little body situated at the base of the skull in connection with the third ventricle of the brain. It is only the size of a small hazel-nut; but it is essential to life, for if it be removed or if its anterior lobe be removed death soon follows. Even partial extirpation of the anterior lobe has serious consequences and leads to atrophy of the organs of generation; while hypertrophy of the gland in youth causes *gigantism*, and hypertrophy in adult life causes *acromegaly*.

During the last few years Professor T. Brailsford Robertson, now of Toronto University, has made many investigations into the chemical nature of the *hormone* of the pituitary body and he has succeeded in extracting from the anterior lobe a chemical substance named by him *tethelin* which has definite and specific effects upon the growth of animals. Professor Robertson found that this substance administered in small doses to growing white mice, at first retarded and afterwards accelerated growth, and ultimately produced animals more compact in form and build than normal animals of the same age fed on ordinary food. Not only so, but the adult animals retained the glossy silky fur of young mice. It was plain, therefore, that tethelin improved general nutrition and especially nutrition of the skin and skin-structures; and experiments were made to discover whether tethelin would hasten recovery of weight in cases of inanition, and whether also it would promote the healing of wounds. It was found that a single hypodermic injection of tethelin accelerated recovery of weight 30 to 50 per cent., and that hypodermic injections at intervals of three days "very markedly accelerated the formation of cicatricial tissue in lesions by excision of small pieces of the skin." In the animals which had received the injections the wounds were completely cicatrised at the end of six days, whereas in the case of untreated animals the wounds usually required ten days to heal. Possibly, therefore, tethelin may be found useful as a means of hastening the healing of wounds, and it may perhaps prove beneficial in tubercular ulcerations, in gastric ulcers, in fractures, in rickets, in osteo-arthritis, in osteomalacia, in senile alopecia, and in various skin diseases.

Dr. Barney writes in the *Journal of Laboratory and Clinical Medicine* of May last: "No work has yet been published on the effects of tethelin as an accelerator of repair in cases of delayed union of fractures, but, reasoning from the basis that acromegaly is caused by hyper-secretion of the anterior lobe of the pituitary, it would appear that tethelin would have a very great value in cases of delayed union. And may we not anticipate less tedious results in the various forms of skin grafting, also in treating gastric ulcers, and, in fact, in almost all epithelial lesions tending to chronicity?" Tethelin may be used either in powder, in ointment, or in aqueous solution. Dr. Robertson seems to have succeeded in isolating a new, interesting, and important hormone, but until we have more evidence we must not be *too* hopeful of its clinical usefulness.

